# The role of Hartmann’s procedure in the elective management of rectal cancer: results of a Brazilian cohort study

**DOI:** 10.1590/0100-6991e-20212977

**Published:** 2021-07-29

**Authors:** ANDERSON RECH LAZZARON, INGRID SILVEIRA, PAULINE SIMAS MACHADO, DANIEL C DAMIN

**Affiliations:** 1 - Hospital de Clínicas de Porto Alegre, Universidade Federal do Rio Grande do Sul, Serviço de Coloproctologia - Pós-graduação em Cirurgia (UFRGS) - Porto Alegre - RS - Brasil

**Keywords:** Rectal Neoplasms, Colorectal Surgery, Postoperative Complications, Neoplasias Retais, Cirurgia Colorretal, Complicações Pós-Operatórias

## Abstract

**Background:**

although preservation of bowel continuity is a major goal in rectal cancer surgery, a colorectal anastomosis may be considered an unacceptably high-risk procedure, particularly for patients with multiple comorbidities. We aimed to assess rates of surgical complications in rectal cancer patients according to the type of procedure they had undergone.

**Materials and Methods:**

this cohort included all rectal cancer patients undergoing elective resection at a referral academic hospital over 16 years. There were three study groups according to the type of performed operation: (1) rectal resection with anastomosis without defunctioning stoma (DS); (2) rectal resection with anastomosis and DS; and (3) Hartmann’s procedure (HP). Postoperative complications and clinical outcomes were assessed*.*

**Results:**

four-hundred and two patients were studied. The 118 patients in group 3 were significantly older (>10 years), had higher Charlson Comorbidity Index scores, and more ASA class ≥3 than patients in the other two groups. Sixty-seven patients (16.7%) had Clavien-Dindo complications grade ≥ III, corresponding to an incidence of 11.8%, 20.9%, and 14.4% in groups 1, 2, and 3, respectively (p=0.10). Twenty-nine patients (7.2%) had major septic complications that required reoperation, with an incidence of 10.8%, 8.2% and 2.5% in groups 1, 2 and 3, respectively (p=0.048). Twenty-one percent of the group 2 patients did not undergo the stoma closure after a 24-month follow-up.

**Conclusion:**

HP was associated with a lower incidence of reoperation due to intra-abdominal septic complications. This procedure remains an option for patients in whom serious surgical complications are anticipated.

## RESUMO

**Introdução:**

embora a preservação do trânsito intestinal seja um dos objetivos principais na cirurgia do câncer retal, a anastomose colorretal pode ser considerada um procedimento de altíssimo risco, particularmente para pacientes com múltiplas comorbidades. Nosso objetivo foi avaliar as taxas de complicações cirúrgicas em pacientes com câncer retal de acordo com o tipo de procedimento a que foram submetidos.

**Materiais e Métodos:**

esta coorte incluiu todos os pacientes com câncer retal submetidos a ressecção eletiva em hospital universitário de referência ao longo de 16 anos. Houve três grupos de estudo de acordo com o tipo de operação realizada: (1) ressecção retal com anastomose, sem estoma desfuncionalizante (ED); (2) ressecção retal com anastomose e ED; e (3) procedimento de Hartmann (PH). Avaliamos as complicações pós-operatórias e os resultados clínicos.

**Resultados:**

estudamos 402 pacientes. O grupo 3 tinha 118 pacientes, estes sendo significativamente mais idosos (>10 anos), com pontuações mais altas no Índice de Comorbidade de Charlson e mais frequentemente classificados como ASA ≥ 3 do que os pacientes dos outros dois grupos. Sessenta e sete pacientes (16,7%) apresentaram complicações de Clavien-Dindo grau ≥ III, correspondendo à incidência de 11,8%, 20,9% e 14,4% nos grupos 1, 2 e 3, respectivamente (p = 0,10). Vinte e nove pacientes (7,2%) apresentaram complicações sépticas graves, necessitando reoperação, com incidência de 10,8%, 8,2% e 2,5% nos grupos 1, 2 e 3, respectivamente (p = 0,048). Vinte e um por cento dos pacientes do grupo 2 não foram submetidos ao fechamento do estoma após acompanhamento de 24 meses.

**Conclusão:**

o PH foi associado à menor incidência de reoperação por complicações sépticas intra-abdominais. Este procedimento continua sendo uma opção para pacientes com alto potencial de desenvolver complicações cirúrgicas graves.

## INTRODUCTION

Anastomotic leak (AL) is still a critical issue in rectal cancer surgery. Despite all recent surgical advances, such as the performance of total mesorectal excision and minimally invasive techniques, the rates of AL remain relatively high (5% to 19%)^1^, with negative impact on morbimortality^2^ ^-^ ^4^, and cancer recurrence^5^. 

The primary method to prevent anastomotic dehiscence after a low anterior resection is to create a DS. Although this strategy may not reduce the incidence of leaking, it can mitigate its consequences, reducing the need for urgent abdominal reoperation^6^. The systematic use of a DS, however, remains controversial, in part because many patients with a “temporary” stoma will never undergo stoma reversal. According to a meta-analysis of ten studies, including 8,568 rectal cancer patients, the nonclosure rate of DSs is 19%^7^. Three variables were significantly associated with nonclosure: older age, ASA score >2, and presence of comorbidities. 

Several risk factors for AL have been identified, including systemic conditions such as anemia, diabetes mellitus, and hypoalbuminemia. Local factors have also been implicated, including irradiation of bowel, intestinal ischemia, and a more distal location of the rectal tumor. So, the crucial decision of performing or not a colorectal anastomosis, particularly in elderly individuals, should take into account the general clinical condition of the patient, including comorbidities and capability to overcome the life-threatening consequences of an AL^8^. 

The management of rectal cancer patients should include a thorough preoperative discussion with the patient and family about the potential risk of AL, its consequences, and the possibility of having a permanent stoma at the end of the treatment. A more conservative approach, such as the performance of a Hartmann procedure, which does not include the construction of a colorectal anastomosis, can be alternatively considered in the critical cases. 

To this date, despite the extensive literature on the surgical treatment of rectal cancer, very few studies have investigated the role of HP in the routine management of patients at a high risk for AL. Our study aimed to assess, in a strictly elective clinical setting, the rates of postoperative complications in patients with rectal cancer according to the type of operations they underwent (with or without colorectal anastomosis) and the presence of risk factors for AL.

## MATERIALS AND METHODS

### Patients and procedures

This retrospective cohort included all patients with rectal adenocarcinoma who underwent elective proctectomy at the Division of Coloproctology between January 1st, 2003, and December 31st, 2018. Medical records were reviewed for demographic, clinical, surgical, and pathological data. All tumors were located up to 15cm from the anal verge. Open, laparoscopic, and robotic tumor-specific mesorectal excisions were performed. All procedures were performed by experienced board certificated colorectal surgeons. When neoadjuvant treatment was used, it was always chemoradiation, using conventional doses of 2 Gy per fraction throughout five to six weeks for a total 50.4 Gy with concurrent 5-fluorouracil-based chemotherapy^9^. Except for the individuals dying of early surgical complications, all patients had a minimum postoperative follow-up of six months.

The exclusion criteria were synchronous distant metastases, palliative surgery, multivisceral resection (pelvic exenteration or partial resection of adjacent organs), and history of primary malignant neoplasm five years before the rectal operations. Abdominoperineal resections of the rectum were also excluded. We did not include patients for whom HP was not initially planned but ended up being performed due to intraoperative complications (such as massive bleeding with hemodynamic instability), which, according to the surgeon, precluded the construction of an anastomosis.

Initially, patients undergoing a colorectal anastomosis were compared with those undergoing a HP. Then, patients were further subdivided in three study groups according to the type of performed operation. Group 1: proctectomy with primary anastomosis without diversion; group 2: proctectomy with primary anastomosis with diversion (transverse colostomy or loop ileostomy); and group 3: HP. All procedures were decided consensually in the preoperative period after extensive discussion with the patient and family considering the potential surgical risks. The type of operation to be performed was routinely registered in the medical record before hospital admission. 

### Assessment of comorbidities

Comorbidities were assessed using the Charlson Comorbidity Index (CCI)10, shown in Chart 1.

**Chart 1:** Charlson Comorbidity Index Scoring System.

Score	Comorbidity
1	Diabetes mellitus without end-organ damage
	Cerebrovascular disease
	Myocardial infarction
	Congestive heart failure
	Peripheral vascular disease
	Dementia
	Chronic pulmonary disease
	Connective tissue disease
	Peptic ulcer disease
	Mild liver disease
2	Diabetes mellitus with end-organ damage
	Moderate/severe renal disease
	Hemiplegia
	Solid tumour without metastasis
	(exclude if > 5 years from diagnosis) leukaemia
	Lymphoma
3	Moderate/severe liver disease
6	Metastatic solid tumour
	AIDS (not just HIV positive)

AL was defined as a “defect of the intestinal wall at the anastomotic site leading to a communication between the intraluminal and extraluminal compartments”. Any abscess near an anastomosis, diagnosed through imaging exams (CT/MRI) or surgical reintervention was considered AL^11^. Patients were also classified according to the American Society of Anesthesiology’s (ASA) Classification System^12^. Tumor staging was determined according to the AJCC TNM Classification of Malignant Tumors, eighth edition^13^.

### Postoperative Complications

Postoperative complications were analyzed according to the Clavien-Dindo (CD) classification system^14^. Grade III, IV, and V complications are the most severe and relevant, being this cutoff point widely used in previous studies^15^ ^,^ ^16^. In the CD system (Chart 2), complications are classified according to how they are managed, not according to etiology.

**Chart 2:** Clavien-Dindo Classification.

Grades	Definition
Grade I	Any deviation from the normal postoperative course without the need for pharmacological treatment or surgical, endoscopic and radiological interventions. Allowed therapeutic regimens include drugs, such as antiemetics, antipyretics, analgesics, diuretics and electrolytes, and physical therapy. This grade also includes wound infections opened at bedside.
Grade II	Requires pharmacological treatment with drugs other than those allowed for grade I complications. Blood transfusions and total parenteral nutrition are also included.
Grade III	Requires surgical, endoscopic or radiological interventions
IIIa	Intervention not under general anaesthesia
IIIb	Intervention under general anaesthesia
Grade IV	Life-threatening complication (including CNS complications*) requiring IC/ICU management
IVa	Single organ dysfunction (including dialysis)
IVb	Multiorgan dysfunction
Grade V	Patient death

*Brain haemorrhage, ischemic stroke, subarachnoid bleeding, but excluding transient ischemic attacks; IC - Intermediate care; ICU - Intense care unit.

We also assessed the incidence of major abdominal septic complications, which included abdominal and pelvic infections (abscess/peritonitis) that required surgical reintervention by laparotomy or laparoscopy. These are the most relevant surgical complications directly resulting from AL or rectal stump leak, representing the main interest of the study. Minor revision procedures, such as pelvic abscess drainage via anal and percutaneous puncture of abdominal collections, or purely mechanical complications (evisceration, bowel obstruction) were not classified as major abdominal septic complications. Finally, in those patients who had a DS, the stoma closure rate was analyzed.

### Sample size

The sample size was calculated based on the study conducted by Jonker et al. The one-tailed Student’s t-test was used to estimate differences in the incidence of major abdominal septic complications between patients with anastomosis and patients without anastomosis (HP). With significance level of 5% and a power of 80%, 392 subjects would be needed.

### Statistical analysis

Pearson’s chi-squared and Fisher’s exact tests were used to determine the association between categorical variables, while ANOVA, Student’s t-test, Kruskal-Wallis, and the Mann-Whitney U test were used to compare the distribution of continuous variables. Categorical variables are presented as frequencies or percentages, and continuous variables are presented as means or medians, depending on the distribution type. Variables independently associated with CD ≥III were determined by the Poisson regression with robust variance. The significance level was set at 5%. This study was approved by our Institutional Ethics Committee under the number 60630116.0.0000.5327.

## RESULTS

A total of 548 patients with rectal cancer underwent proctectomy during the study period. One-hundred fourty-six of them were excluded according to the exclusion criteria, resulting in a study population of 402 patients. Their clinical characteristics are shown in Table 1. Patients who underwent HP (group 3) were significantly older (>10 years), had a higher CCI score, and a higher proportion of ASA class ≥3 than patients in the other two study groups. In contrast, group 2 had more distal tumors, underwent more neoadjuvant therapies, and there was a higher percentage of men. Table 2 shows the incidence of comorbidities between patients undergoing a HP and those who underwent a colorectal anastomosis and protective stoma. The median follow-up of the study was 38 months (interquartile range = 41 months).

**Table 1:** Demographic and clinical characteristics of the patients (N = 402).

Variable	Group 1	Group 2	Group 3	p-value
n (%)	102 (25.3)	182 (45.2)	118 (29.3)	
Age, mean (range), years	59.3 (31-83) a	60.0 (30-82) a	71.0 (43-90) b	< 0.001
Male, n (%)	45 (44.1)	123 (67.6)	67 (56.8)	< 0.001
Tumour height, median (IQR), cm	13 (10-15) a	7 (6-9)b	8 (6-11.5) b	< 0.001
Tumour size***, mean, cm	4.3 (0.5-10) a	3.5 (0.6-14) a	4.8 (0.8-15) b	< 0.001
Charlson Comorbidity Index, median (IQR)	2 (2-3)a	2 (2-3) a	3 (2-4) b	< 0.001
Charlson Comorbidity Index n ≥ 3, n (%)	34 (33.3)	46 (25.3)	81 (68.6)	< 0.001
ASA Class ≥ 3 (%), n	6 (5.9)	14 (7.7)	38 (32.2)	< 0.001
BMI, mean (range), Kg/m^2^	26.6 (18-39.7)	25.6 (17.6-39)	25.3 (15.6-41.2)	0.401
Neoadjuvant therapy****	12 (11.8)	91 (50.3)	28 (23.7)	< 0.001
Albumin**, mean, mg/dl	4.2 (2.8-5.0) a	4.1 (2.3-4.9) a	3.9 (3.0-4.8) b	< 0.001
Haemoglobin**, mean, mg/dl	12.9 (6.9-16.4) a	12.6 (8.1-17.9) a	11.7 (8.5-14.4) b	< 0.001

ASA - American Society of Anaesthesiology classification; BMI - Body mass index; CEA - Carcinoembryonic antigen; IQR - Interquartile range;

+ Significant according to adjusted residual analysis; ** Preoperative examination; *** Pathological measurement; **** Four missing; Letter

system (different letters) - Statistically significant difference.

**Table 2:** Differences in comorbidities between patients in groups 2 and 3.

Comorbidities	Group 2 (n=182)	Group 3 (n=118)	p-value*
Myocardial infarction, n (%)	2 (1.0)	19 (16.1)	<0.001
Heart failure, n (%)	6 (3.2)	12 (10.1)	0.027
Chronic obstructive pulmonary disease, n (%)	15 (8.2)	38 (32.2)	<0.001
Cerebrovascular accident, n (%)	1 (0.5)	4 (3.3)	0.15
Dementia, n (%)	1 (0.5)	2 (1.6)	0.7
Diabetes mellitus, n (%)	16 (8.7)	32 (27.1)	<0.001
Kidney disease, n (%)	4 (2.1)	9 (7.6)	0.024
Liver disease, n (%)	4 (2.1)	8 (6.7)	0.047
Hemiplegia, n (%)	0 (0)	2 (1.6)	0.15**

* Chi-square with Yates correction.

** Fisher’s exact test. All others: Chi-square with Yates correction.

### Postoperative complications according to Clavien-Dindo classification

Of the 402 patients, 67 (16.7%) had severe postoperative complications (CD grade ≥ III), as shown in Tables 3 and 4. The incidence of CD grade ≥ III was 11.8%, 20.9% and 14.4% in groups 1, 2 and 3, respectively (p = 0.10). Univariate analyses were performed to determine which variables were independently associated with CD grade ≥ III. The following variables were selected for the multivariate analysis: male sex (RR = 1.93 - p = 0.01), distal tumor location (p = 0.006), and neoadjuvant therapy (RR = 1.82 - p = 0.009). Poisson regression with robust variance was then performed with these three variables: male sex (RR = 1.85 - 95% CI 1.12 - 3.04; p = 0.015) and low tumor location (RR = 1.10 - 95% CI 1.03 - 1.18; p = 0.004) remained significantly associated with CD grade ≥ III.

**Table 3:** Clavien-Dindo grade ≥ III complications and management (n=67).

Complication	Management
Group 1 (n=12)	
Anastomosis dehiscence/peritonitis - 11 cases*	laparotomy - 11 cases*
Intestinal obstruction by adhesion - 1 case	laparotomy - 1 case
Group 2 (n = 38)	
Duodenal ulcer/upper gastrointestinal bleeding - 1 case	endoscopic management - 1 case
Postoperative abdominal bleeding - 2 cases	laparotomy - 2 cases
Intestinal obstruction - 3 cases	laparotomy - 3 cases
Evisceration - 1 case	abdominal wall resuturing - 1 case
Severe stoma prolapse - 1 case	local approach without laparotomy - 1 case
Abscess/ abdominal or pelvic peritonitis - 30 cases	laparotomy - 15 cases* percutaneous drainage - 15 cases
Group 3 (n = 17)	
ARDS after pulmonary aspiration - 1 case Sepsis of pulmonary origin - 2 cases	ICU management - 1 case ICU management - 2 cases
Pelvic abscess - 4 cases	anal drainage - 3 cases laparotomy - 1 case*
Abscesses/peritoneal collections - 4 cases	percutaneous drainage - 2 cases laparotomy - 2 cases*
Evisceration - 4 cases	resuturing abdominal wall - 4 cases
Dehiscence/colostomy necrosis - 2 cases	local approach without laparotomy - 2 cases

* Major septic complication (relaparotomy); ARDS - Adult respiratory distress syndrome; ICU - Intensive care unit.

**Table 4:** Postoperative complications according to the study groups.

Complications*	Group 1	Group 2	Group 3	p-value
n (%)	102 (25.3)	182 (45.2)	118 (29.3)	
Clavien-Dindo grade ≥ III	12 (11,8)	38 (20,9)	17 (14,4)	0,10
Major septic complications	11 (10.8)	15 (8.2)	3 (2.5)	0.048

* Complications were measured within 30 days.

+ Significant according to adjusted residual analysis.

### Major abdominal septic complications

Twenty-nine patients (7.2%) had major abdominal septic complications. When all patients with anastomosis (groups 1 and 2) were compared with those who underwent HP, the incidences of major septic complications were 9.1% and 2.5%, respectively (p = 0.034). When the patients were further subdivided in the three study groups, the incidence was 10.8%, 8.2% and 2.5% in Groups 1, 2 and 3, respectively (p = 0.048). When only patients with CCI ≥3 were analyzed, this difference was even more marked (Table 5). While 7 patients with CCI ≥3 in group 2 (15.2%) had major abdominal septic complications requiring abdominal reoperation, none in the Hartmann group presented this complication (< 0.001).

**Table 5:** Postoperative complications and mortality among CCI ≥ 3 patients (n = 161).

Outcome	Group 1	Group 2	Group 3	p-value
n (%)	34 (21.1)	46 (28.6)	81 (50.3)	
CCI ≥ 3	4 (11.8)	10 (21.7)	16 (19.7)	0.49*
Major septic complications	3 (8.8)	7 (15.2)	0 (0)	<0.001*
Mortality	1 (2.9)	1 (2.2)	3 (3.7)	1.0*

* Freeman-Halton extension of Fisher’s exact test.

Of the 284 patients who had anastomosis, AL was diagnosed in 51 (17.9%). There were 14 AL cases (13.7%) in group 1 and 37 AL cases (20.3%) in group 2. Overall, the 30-day mortality was 2.9%, 1.1% and 3.4% in Groups 1, 2 and 3, respectively (p = 0.31).

### Proctectomies with a protective stoma

A total of 182 patients had a DS. We analyzed all patients within a minimum postoperative follow-up of 24 months, resulting in 147 patients. Of these, 115 (78.2%) underwent stoma reversal. In three cases, the stoma had to be constructed again: two due to AL after the reversal operation, and one because of severe fecal incontinence (rediversion required after 14 months). The reasons why the other 32 patients (21.8%) did not undergo the reversal operation are presented in Table 6. Table 7 has the information regarding patients who did or did not undergo reversal surgery. The median time for the reversal was 12 months (interquartile range = 8 months).

**Table 6:** Reasons for not performing the stoma closure (n=32).

Reason	n
Patient option (refusal)	7
Anastomotic problems	12
Stenosis	6
Pre-sacral sinus	2
AL in initial surgery	2
Rectal vaginal fistula	2
Progression of neoplasia	6
Poor clinical conditions	3
Pulmonary embolism	1
Perforation of small bowel due to actinic enteritis	1
Primary lung cancer	1
Serious complications in the initial surgery (hostile abdomen)	2
Investigation of possible recurrence	2
Suspected pulmonary nodules	1
Retroperitoneal nodule next to the iliac artery	1

Note: follow-up in all cases ≥ 24 months.

**Table 7:** Characteristics of patients in group 2 (24-month follow-up, n = 147) according to the reversal of the stoma.

Variable	Closure of the stoma	Nonclosure of the stoma	p-value
n (%)	115 (78.2)	32 (21.8)	-
Age, mean (range), years	59.3 (30-79)	60.9 (34-79)	0.45
Male, n (%)	81 (70.4)	17 (53.1)	0.10
Tumour height, median (IQR), cm	8 (7-9)	7 (5-8)	0.042
CEA, median (IQR), mg/dL	2.9 (1.4-7.0)	2.5 (1.6-10.2)	0.79
Resected lymph nodes, median (IQR), n	17 (14-24)	18 (13.2-21.7)	0.62
Tumour size***, median (IQR), cm	3.6 (2.2-5.3)	3.2 (2.3-4.5)	0.25
Charlson Comorbidity Index, median (IQR)	2 (2-2)	2 (2-3)	0.16
Charlson Comorbidity Index ≥3, n (%)	23 (20)	10 (31.3)	0.26
ASA class ≥ 3 (%), n	11 (9.6)	1 (3.1)	0.46
BMI, mean (range), Kg/m^2^	25.7 (17.6-39)	25.4 (18.9-34.2)	0.78
Neoadjuvant therapy (%), n	49 (42.6)	18 (56.3)	0.25
Pathological stage, (%), n			
0	6 (5.2)	5 (15.6)	0.16
I	32 (27.8)	6 (18.8)	
II	37 (32.2)	8 (25)	
III	40 (34.8)	13 (40.6)	
Albumin**, median (IQR), mg/dL	4.2 (3.9-4.4)	4.1 (3.9-4.3)	0.25
Haemoglobin**, mean (range), mg/dL	12.7 (8.5-17.9)	12.3 (8.1-15.6)	0.29
Anastomotic leak, n (%)	17 (14.8)	7 (21.9)	0.50
Tumour recurrence****, n (%)	24 (20.9)	12 (37.5)	0.089

ASA - American Society of Anaesthesiology classification; BMI - Body mass index; CEA - Carcinoembryonic antigen; IQR - Interquartile range;

** Preoperative examination; *** Pathological measurement; **** Local and/or systemic recurrence.

## DISCUSSION

The current article approaches one of the most crucial dilemmas of the colorectal surgeon: to perform or not an anastomosis in a patient with a high risk for AL. Our study is, to our knowledge, the first to compare surgical results between the preoperatively planned HP and the colorectal anastomosis in patients undergoing elective rectal resections.

Due to the observational nature of the study, a retrospective cohort, there was a series of significant differences between the study groups. As expected, patients undergoing HP were older, had higher CCI scores and ASA classes, reflecting the selective decision regarding rectal anastomosis our team has adopted over the years. The high proportion of HP in our series (29.3%) seems to be related with the low socioeconomic status of the study population. All the patients were using the governmental health system, and they usually have only limited access to specialized health services and proper diagnostic checkups. As a consequence, many of them present with more advanced tumors and multiple poorly managed comorbidities. Despite their significantly worse clinical conditions, patients in group 3 did not present increased CD ≥III complications. This result suggests that the strategy of avoiding an anastomosis in patients with multiple comorbidities might have reduced their chance of presenting severe postoperative complications. As previously demonstrated, higher ASA scores have been associated with increased postoperative morbidity, including a higher incidence of AL^17^.

We also decided to analyze a second primary endpoint: major septic abdominal complications, which included abdominal and pelvic infections (abscess/peritonitis) that demanded surgical reintervention, representing the most relevant complications directly related either with AL or rectal stump leak. Patients in the Hartmann group had a 2.5% complication rate compared with 9.1% in the other two groups (p = 0.034, RR = 0.27), and they required 3.6 times fewer relaparotomies. 

Our results are in line with the study conducted by Sverrisson et al.^18^, who retrieved data from the Swedish Colorectal Cancer Registry for patients operated on for rectal cancer between 2007 and 2014. Of 10,940 patients, 1,452 (13%) underwent HP (median age 77 years). The ASA score was 3-4 in 43% of the patients, and 15% had distant metastases. Overall and surgical complication rates were 41% and 26%, respectively. The incidence of abdominal infections was 8%, and the relaparotomy rate was 10%. The authors concluded that, despite older age and comorbidities, including more advanced cancer, patients undergoing HP had a low incidence of serious complications. However, they reported a high frequency of intraoperative bowel perforation (8%), which, as they recognized, could be the reason for the surgeon to perform the HP.

Jonker et al.^19^ conducted a retrospective study based on the Dutch National Cancer Registry to compare the outcome after HP and low anterior resection with or without a diverting ileostomy in patients with rectal cancer who received neoadjuvant radiotherapy. A total of 4,288 patients were included: 27.8% underwent HP, 20.2% low anterior resection, and 52.0% low anterior resection with DS. Patients in whom HP was performed were significantly older, had more comorbidities, and were more often classified as ASA 3 or 4. Thirty-day mortality was higher after HP (3.2% vs. 1.3% and 1.3% for low anterior resection with or without DS, p<0.001), but HP was not an independent predictor of mortality in the multivariable analysis. HP and low anterior resection with DS were associated with a lower rate of intra-abdominal infections (6.5% and 10.1% vs.16.2%, p<0.001) and reoperations (7.3% and 8.1% vs.16.5%, p<0.001). HP also had the lowest rate of an endpoint described as “any postoperative complication”.

Our study is distinct from the previous reported studies because we only analyzed patients operated on with a curative intent for whom the type of operation (with or without anastomosis) was defined preoperatively. Patients for whom HP was not initially planned but was performed due to intraoperative complications (such as massive bleeding, hemodynamic instability or perfuration) were excluded from the analysis since their procedures are usually longer and technically more complex. So, we analyzed the results of the three different operations in a strictly elective setting.

The AL rate in Groups 1 and 2 was 17.9%. Previous studies have found post-proctectomy AL rates ranging from 5 to 19%^1^ ^,^ ^20^. Our relatively high rate of AL can be explained, in part, by our rigid definition of AL, which included any perianastomotic collection or abscess^11^. Also, we routinely follow a strict protocol of AL detection, which included C-reactive protein measurement on the fourth postoperative day, followed by abdominal CT-scan whenever the protein level was elevated. With this sequence, we can eventually detect pelvic abscesses that result from infected hematomas or intraoperative contamination, not from a true AL. 

Another relevant aspect of our study is the analysis of the patients who had a DS. After 24 months, about 22% of patients did not have the stoma reversed. The only factor significantly associated with the nonreversal was a more distal tumor location. Previous authors have identified specific characteristics that reduce the chance of closing temporary stomas. Pan et al.^21^ investigated 296 patients who underwent anterior resection of the rectum with protective ileostomy. After a mean follow-up of 29 months, the ileostomy could not be closed in 17.2% of the patients. Metastatic disease, a CCI score >1, and complications during the initial operation were independent risk factors for non-reversal of ileostomies. More recently, a meta-analysis aimed to identify the risk factors associated with the nonclosure of DS after rectal cancer surgery^7^. Ten studies with 8,568 patients were reviewed. The nonclosure rate was 19%. Three demographic factors were associated with nonclosure: older age, ASA score >2 and comorbidities. Besides, surgical complications, AL, stage IV tumor, and local recurrence were strong risk factors for nonclosure.

One issue that should not be underestimated is the morbidity of the stoma closure^15^ ^,^ ^22^. A meta-analysis assessed 6,107 patients undergoing the closure of loop ileostomy. Overall morbidity was 17.3%, with a mortality rate of 0.4%. Almost 4% of patients required a laparotomy to close the ileostomy. The most common postoperative complication was small bowel obstruction (7.2%)^23^.

Another important point to be considered is the chance of developing low anterior resection syndrome (LARS), a condition known for its highly negative impact on patients’ quality of life. A recent meta-analysis of 11 studies found that the estimated prevalence of major LARS was 41%. Radiotherapy and low tumor height were the most consistently assessed variables, both presenting a negative effect on bowel function. DS was found to have a significant negative impact on bowel function in 4 of 11 studies^24^.

We strongly believe that the type of operation to be performed in rectal cancer should be decided preoperatively. Patients with multiple comorbidities must be informed about their increased risk of AL and the severe associated consequences, including a higher chance of undergoing surgical reintervention and death. They also need to know that, when a DS is created, mainly when the tumor is more distally located, there will be about a 20% chance of nonclosure of the stoma. So, under that circumstance, HP may be discussed as a valid surgical option.

Our study has the intrinsic limitations of a retrospective series, which explains the clinical differences observed between the study groups. Even though all procedures were performed by well-trained colorectal surgeons, the experience of each of the surgeons, as usually occurs in surgical series, might be different. Similarly, some differences in the use of open and minimally invasive techniques might have some influence in the surgical results.

## CONCLUSION

Patients with rectal cancer undergoing HP had a lower incidence of intra-abdominal septic complications that resulted in abdominal reoperation. About 20% of the patients who had a DS did not have the stoma reversed. HP can be considered valid option for frail patients with multiple comorbidities.
